# National trends, disparities and forecasts in substance use disorder–related suicide mortality in the United States: a CDC WONDER analysis

**DOI:** 10.3389/fpubh.2026.1830159

**Published:** 2026-06-18

**Authors:** Tuo Zhang, Yi Shen, Qiong Chen, Junwei Du, Tianshu Hou

**Affiliations:** 1 Traditional Chinese Medicine Prevention and Treatment Center, Chengdu Integrated Traditional Chinese Medicine and Western Medicine Hospital, Chengdu, China; 2 School of Acupuncture and Tuina, Chengdu University of Traditional Chinese Medicine, Chengdu, China

**Keywords:** CDC WONDER, disparities, substance use disorder, suicide, trends

## Abstract

**Background:**

Suicide remains a leading cause of mortality in the United States, and substance use disorders (SUD) are strong predictors of suicide risk. Long-term trends and disparities in SUD-related suicide mortality remain insufficiently characterized.

**Methods:**

We analyzed 2001–2023 mortality data from the CDC WONDER Multiple Cause of Death database, using the most recent complete 23-year national time series. SUD-related suicide deaths were defined as deaths with suicide as the underlying cause (ICD-10 U03, X60–X84, Y87.0) and SUD listed among multiple-cause or contributing conditions (ICD-10 F10–F19). Each death was counted once. AAMRs were calculated by sex, race/ethnicity, census region, and urban-rural status, while age-specific rates were calculated by age group. Joinpoint regression estimated APC and AAPC. ARIMA and ETS models forecasted AAMRs through 2035.

**Results:**

The overall AAMR increased from 0.45 in 2001 to 0.74 in 2023, representing a 64% rise (AAPC, 1.70, 95% CI, 1.16–2.24). Men consistently had higher mortality than women, whereas women showed steeper proportional increases. Mortality was highest among adults aged 45–54 years and lowest among those aged 15–24 years. Non-Hispanic White individuals, the Midwest, and nonmetropolitan areas had the highest burdens. ARIMA projected largely stable rates with widening prediction intervals, whereas ETS suggested a modest increase through 2035, mainly driven by the male series.

**Conclusions:**

SUD-related suicide mortality increased substantially and remained unevenly distributed across demographic and geographic groups. Divergent forecasts highlight uncertainty and the need for targeted prevention.

## 1 Introduction

Suicide is a predominant cause of violent death worldwide, accounting for 1.5% of all fatalities (1), and it has significant social and emotional repercussions for individuals, families, and communities (2). Although global suicide rates have substantially decreased in recent decades, the United States continues to record concerning statistics. In 2017, suicide ranked as the tenth largest cause of death in the United States, accounting for over 47,000 fatalities. Between 1999 and 2017, suicide rates rose by 33%, culminating in a peak of 14 fatalities per 100,000 individuals (3). This trend highlights the critical necessity for efficient preventative and intervention efforts.

Substance Use Disorder (SUD), encompassing alcohol use disorder (AUD), cannabis use disorder (CUD), and drug use disorder (DUD), is a significant risk factor for suicide (4, 5). SUDs are significantly associated with an increased risk of suicidal behavior (6). Research indicates that roughly 25–50% of all suicides are associated with substance use disorders, with alcohol consumption accounting for 22% of these instances (7). SUDs constitute the second most prevalent cause (22.4%) of suicides in outpatient settings, with a nearly two-fold increase in inpatient settings (8). Approximately 40% of individuals with substance dependence who pursue treatment have a history of at least one suicide attempt (9). Recent data indicate that cannabis use may be associated with suicidal behavior (10), with frequency of use correlated with a heightened incidence of suicide attempts (11). Individuals with SUDs are at elevated risk of suicide even after accounting for concomitant psychiatric diseases such as depression (12). The association between substance use and suicide is complex; substance use may be linked to increased impulsivity, disrupted social connections, and impaired decision-making, which may contribute to vulnerability to suicidal behavior.

Although the association between SUD and suicide is well documented, national mortality patterns for SUD-related suicide remain insufficiently characterized. The unequal distribution of this burden across demographic and geographic groups complicates risk assessment and highlights the need for comprehensive surveillance. To address this gap, we used the CDC WONDER database to examine SUD-related suicide mortality trends from 2001 to 2023 by age, sex, race/ethnicity, census region, and urban-rural classification. The study period was selected because 2023 was the most recent complete mortality year available for this analysis and because it provided a 23-year ICD-10-era time series, including the COVID-19 period rather than excluding it. To provide a forward-looking perspective, we also applied ARIMA and ETS models to generate model-specific forecasts through 2035. Understanding these patterns may support targeted prevention and resource allocation for high-burden populations.

## 2 Materials and methods

### 2.1 Data extraction

This research used mortality data generated by the National Center for Health Statistics (NCHS) and accessed through the Centers for Disease Control and Prevention's Wide-Ranging Online Data for Epidemiologic Research (CDC WONDER) Multiple Cause of Death database (13). Data are updated annually from death certificates of U.S. residents and include the underlying cause of death, multiple contributing causes, and demographic information. We analyzed deaths from 2001 through 2023, the most recent complete period available for this analysis. SUD-related suicide was defined as a death with suicide listed as the underlying cause of death (ICD-10 codes U03, X60-X84, or Y87.0) and SUD listed among the multiple-cause/contributing conditions (ICD-10 codes F10–F19). Thus, SUD was not required to be the underlying cause of death. Deaths for which SUD was the underlying cause and suicide appeared only as a contributing condition were not included. Each eligible death certificate was counted once, which avoided double-counting when both suicide and SUD codes were present on the same record. Prior studies have used these ICD-10 categories to identify suicide and SUD within administrative datasets (14, 15). Crude mortality rates (CMRs), age-specific mortality rates, and age-adjusted mortality rates (AAMRs) per 100,000 population, together with corresponding confidence intervals (CIs) and standard errors (SEs), were obtained. AAMR was standardized to the 2000 U.S. standard population. Mortality rates were retrieved by sex, age group, race/ethnicity, census region, and urban-rural classification. Age categories were 15–24, 25–34, 35–44, 45–54, 55–64, and 65–74 years. Restricting the analysis to individuals aged 15–74 years was intended to improve the stability and comparability of mortality estimates across calendar years and demographic strata while capturing the main age-related patterns of SUD-related suicide mortality. Racial/ethnic categories included Hispanic, Non-Hispanic (NH) Black, NH White, and NH other groups (including NH Asian or Pacific Islander, NH Hawaiian, and NH American Indian or Alaska Native, among others). Urban and rural categories were based on the National Center for Health Statistics Urban-Rural Classification Scheme, which distinguishes metropolitan counties from nonmetropolitan counties according to the 2013 U.S. census classification (16). The U.S. Census Bureau regional divisions were categorized as Northeast, Midwest, South, and West (17). The study complied with the Strengthening the Reporting of Observational Studies in Epidemiology (STROBE) principles to enhance transparency and scientific rigor in observational research (18).

### 2.2 Statistical analysis

The Joinpoint Regression Program version 5.1, supplied by the National Cancer Institute (NCI), was used to examine mortality patterns from 2001 to 2023 (19). This program detects changes in AAMR and estimates APC using log-linear regression when temporal variation is present. The number of joinpoints was determined by the Monte Carlo permutation method, which identifies the statistically optimal number of trend segments. Confidence intervals (CIs) at 95% for AAMRs were computed using standard error estimates supplied by the Joinpoint Regression Program. APC and AAPC were computed for each time segment and for the entire study period. AAPC was calculated as a weighted average of segment-specific APCs to summarize the mortality trend over the full study duration. APCs and AAPCs were classified as increasing or decreasing if the slope differed significantly from zero on a two-tailed *t*-test. Statistical significance was established at *p* <= 0.05. Statistically significant values are denoted with an asterisk (^*^) in the Results section.

To project future SUD-related suicide mortality in the United States, we used autoregressive integrated moving average (ARIMA) and error-trend-seasonality/exponential smoothing state space (ETS) models as complementary forecasting approaches rather than pooled or averaged estimates. ARIMA models can account for underlying trends, autocorrelation, seasonality, and nonstationarity through autoregressive, moving-average, and differencing components (20). ETS models use exponential smoothing to decompose a time series into error, trend, and seasonal components and generate forecasts by weighting historical observations rather than explicitly modeling lagged autoregressive terms (21). Because random-walk ARIMA specifications without drift produce flat multi-step point forecasts by construction, ARIMA and ETS forecasts were interpreted separately. Forecasting analyses were restricted to the overall and sex-specific series to enhance interpretability and stability of long-horizon projections and to avoid unreliable extrapolation in sparsely populated subgroups with substantial year-to-year variability. All time-series modeling and forecasting were conducted in R using the forecast package.

AAMRs from 2001 through 2023 were ordered chronologically and modeled as nonseasonal time series. ARIMA specifications were identified using auto.arima(), with model fit and parsimony summarized using information criteria (AIC, AICc, and BIC). Model adequacy was assessed via residual diagnostics, including the Ljung-Box test for remaining autocorrelation and inspection of residual autocorrelation patterns. When diagnostics were acceptable, fitted ARIMA models were used to generate forecasts of AAMR for 2024–2035 with 95% prediction intervals. ETS models were fitted as a prespecified supplementary sensitivity analysis using automated model selection, with information criteria (AICc and BIC) recorded and residual behavior evaluated using Ljung-Box testing and residual autocorrelation diagnostics. Forecasts were reported by model to avoid implying a single combined projection.

## 3 Results

The overall AAMR increased from 0.45 to 0.74 between 2001 and 2023, indicating a 64% increase in SUD-related suicide mortality (AAPC: 1.70; 95% CI: 1.16–2.24). A distinct age gradient was observed, with rates highest among adults aged 45–54 years (0.92) and lowest among those aged 15–24 years (0.28). Men consistently showed higher mortality than women (0.88 vs. 0.40). Non-Hispanic Black individuals had the lowest AAMR (0.21), while Non-Hispanic White individuals had the highest AAMR (0.86), followed by Non-Hispanic Other and Hispanic groups. The Northeast had the lowest burden (0.37), whereas the Midwest had the highest (0.88). Nonmetropolitan areas had greater AAMRs than metropolitan areas during the available urbanization analysis period (0.91 vs. 0.59), and the gap widened after 2011.

### 3.1 Annual trends in SUD-related suicide AAMR

**Figure 1:**
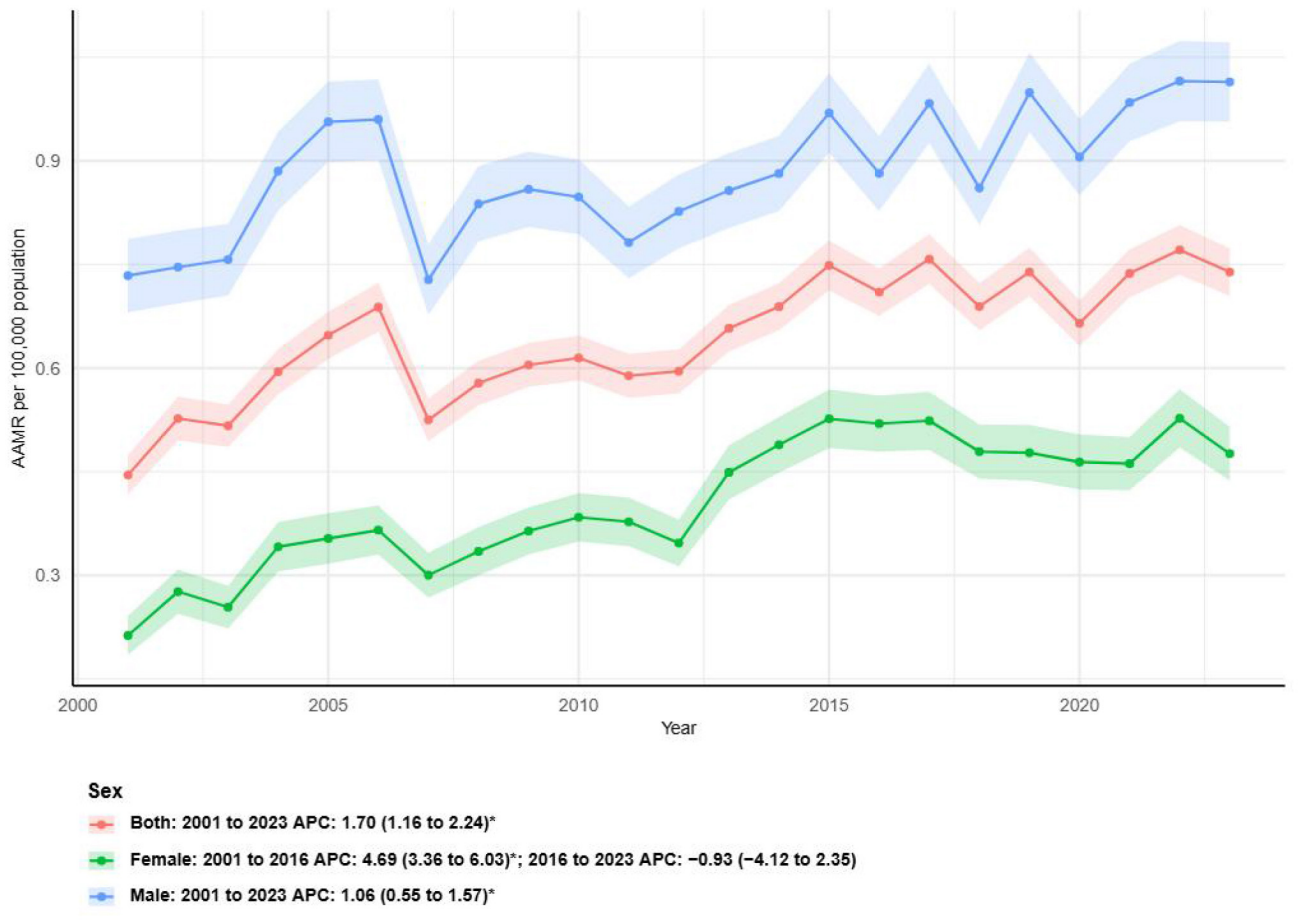
Overall and sex-stratified SUD-related suicide AAMRs per 100,000 in the United States, 2001–2023. * Indicates that the annual percentage change (APC) is significantly different from zero at α = 0.05. AAMR, Age-adjusted mortality rates.

Between 2001 and 2023, the overall AAMR for SUD-related suicide increased from 0.45 to 0.74, representing a significant long-term rise (AAPC: 1.70; 95% CI: 1.16–2.24). The trend was characterized by a sharp increase in the early 2000s (APC: 6.12; 95% CI: 2.84–9.52 for 2001–2005), followed by a period of relative stability through the late 2000s and mid-2010s (APC: 0.47; 95% CI: −0.65 to 1.59 for 2005–2015), and a renewed significant incline thereafter (APC: 2.29; 95% CI: 1.05–3.55 for 2015–2023), culminating in nearly double the annual deaths by the end of the study period (Figure 1, Supplementary Tables 1, 2).

### 3.2 SUD-related suicide AAMR stratified by sex

During 2001–2023, males consistently had higher AAMRs than females (0.88 vs. 0.40). Among men, the AAMR increased from 0.73 in 2001 to 1.01 in 2023, with a modest but significant long-term rise (APC: 1.06; 95% CI: 0.55–1.57). In contrast, women had lower baseline rates but a steeper relative increase: from 0.21 in 2001, the AAMR rose rapidly until 2016 (APC: 4.69; 95% CI: 3.36–6.03), reaching a peak of 0.54, followed by a slight, non-significant decline through 2023 to 0.48 (APC: −0.93; 95% CI: −4.12 to 2.35). Overall, while men bore the higher absolute burden, women demonstrated faster proportional growth over the study period (Figure 1, Supplementary Tables 1, 2).

### 3.3 Age-specific SUD-related suicide mortality rates stratified by age group

**Figure 2:**
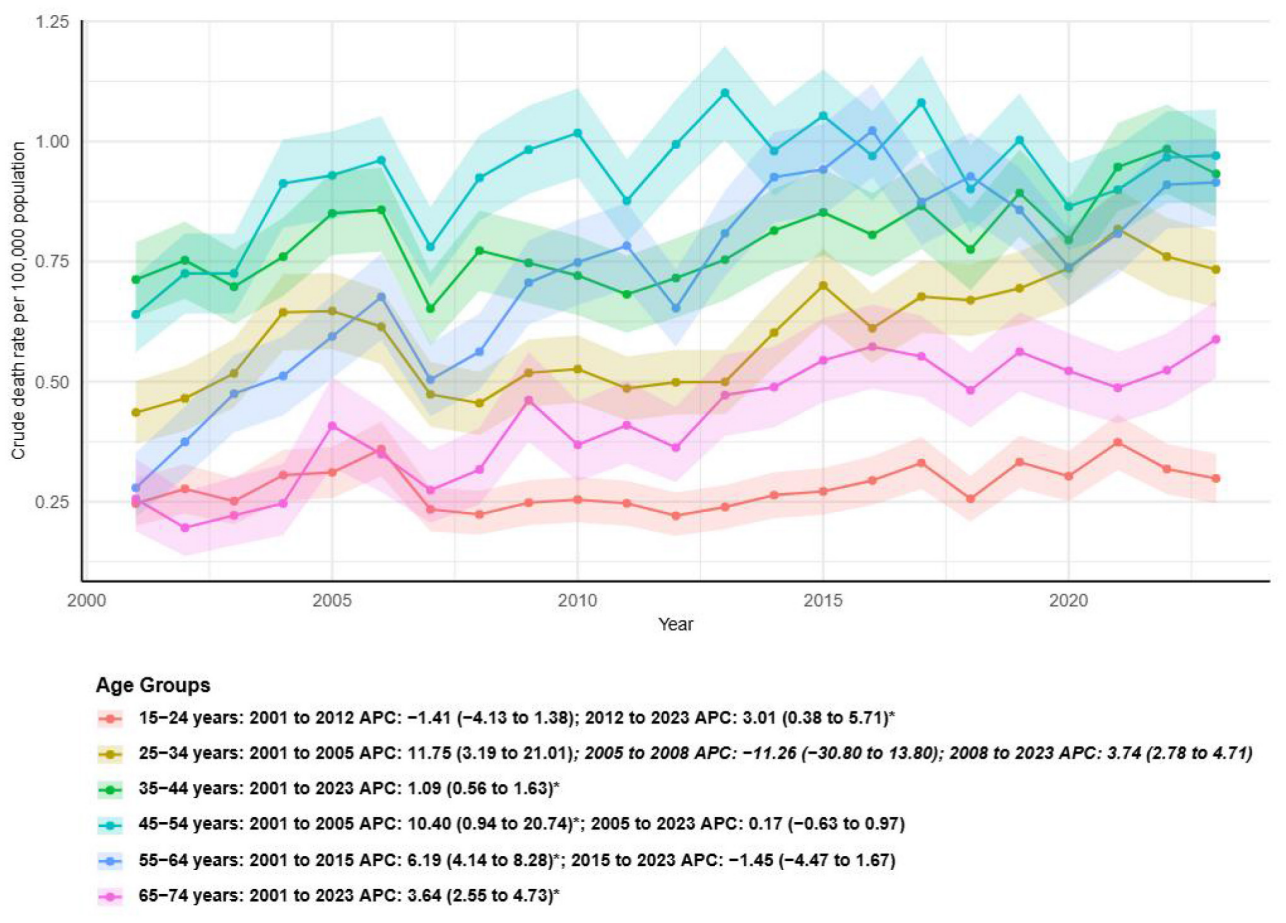
Age-specific SUD-related suicide mortality rates per 100,000 in the United States, 2001–2023. * Indicates that the annual percentage change (APC) is significantly different from zero at α = 0.05.

When stratified by age group, mortality rates were highest among adults aged 45–54 years (0.92), followed by those aged 35–44 years (0.80), 55–64 years (0.72), 25–34 years (0.60), and 65–74 years (0.42), while the lowest rates were observed among individuals aged 15–24 years (0.28). Patterns over time varied markedly by age. People aged 15–24 years experienced a slight decline early in the period (APC: −1.41% from 2001 to 2012) followed by a significant recent increase (APC: 3.01% from 2012 to 2023). Those aged 25–34 years showed a sharp rise in the early 2000s (APC: 11.75% during 2001–2005), a brief nonsignificant dip around 2005–2008, and then a continued significant increase thereafter (APC: 3.74% from 2008 to 2023). The 35–44-year age group had a modest but steady mortality increase over the full study period (APC: 1.09% from 2001 to 2023). Adults aged 45–54 years had a large initial increase (APC: 10.40% from 2001 to 2005), followed by essentially stable rates from 2005 to 2023. The 55–64-year group showed a prolonged significant rise until 2015 (APC: 6.19% from 2001 to 2015), then plateaued thereafter with no significant trend. Finally, adults aged 65–74 years experienced a steady significant increase throughout 2001–2023 (APC: 3.64%) (Figure 2, Supplementary Tables 3, 4).

### 3.4 SUD-related suicide AAMR stratified by race/ethnicity

**Figure 3:**
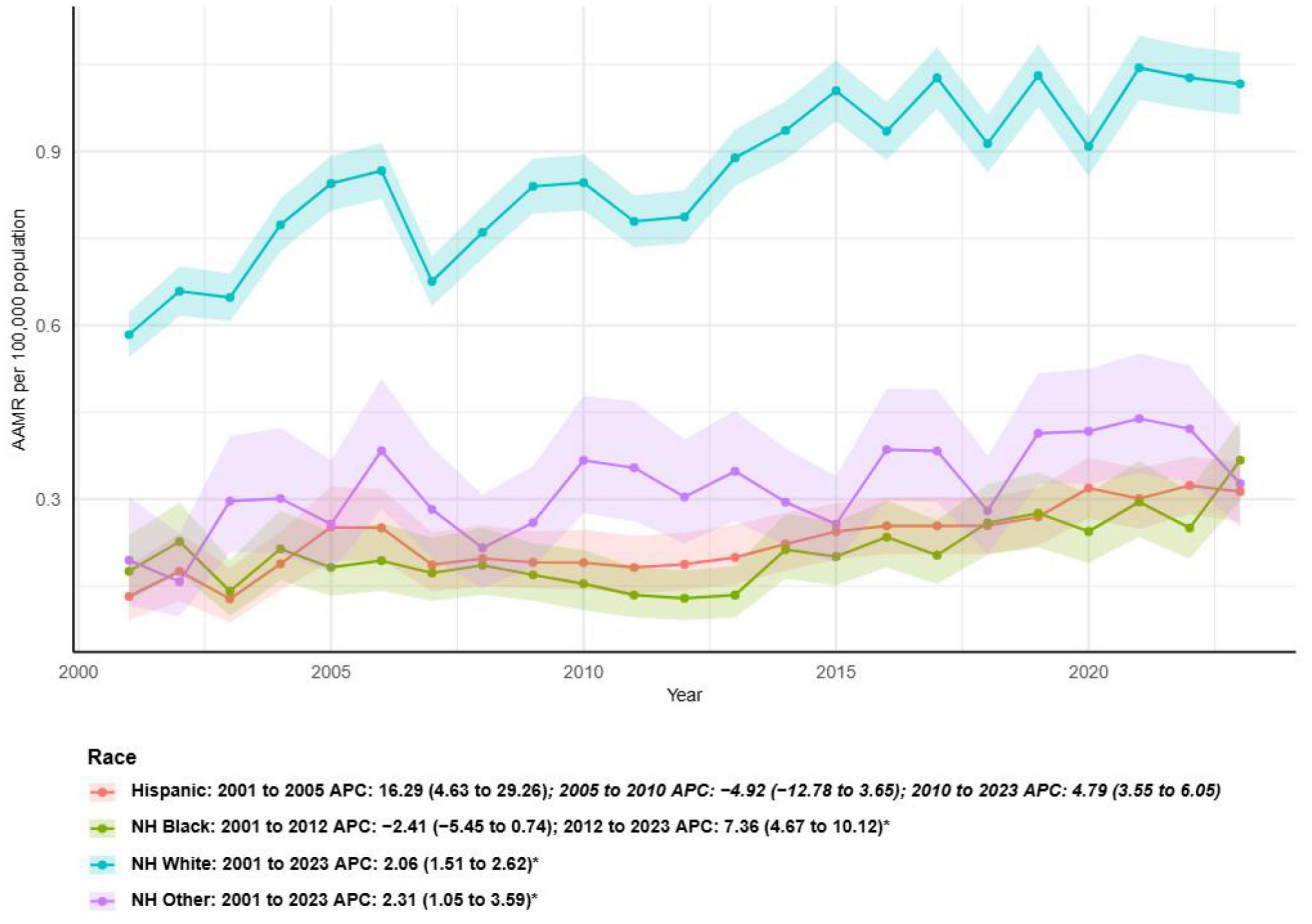
Race/ethnicity-stratified SUD-related suicide AAMRs per 100,000 in the United States, 2001–2023. * Indicates that the annual percentage change (APC) is significantly different from zero at α = 0.05. AAMR, Age-adjusted mortality rates.

By race/ethnicity, Non-Hispanic White adults had the highest AAMR (0.86), followed by Non-Hispanic Other (0.32) and Hispanic (0.23), while Non-Hispanic Black populations had the lowest rates (0.21). Among Hispanic populations, the AAMR rose modestly in the early 2000s, followed by a significant increase after 2010 (APC: 4.79; 95% CI: 3.55–6.05). Non-Hispanic Black populations remained relatively stable from 2001 to 2012 (APC: −2.41; 95% CI: −5.45 to 0.74), but subsequently demonstrated a sharp significant rise from 2012 to 2023 (APC: 7.36; 95% CI: 4.67–10.12). The AAMR trend for non-Hispanic White populations was characterized by a consistent and significant increase across the study period (APC: 2.06; 95% CI: 1.51–2.62), making them the group with the highest sustained burden. Non-Hispanic Other populations also experienced significant long-term growth (APC: 2.31; 95% CI: 1.05–3.59), with rates rising steadily from 2001 through 2023 (Figure 3, Supplementary Tables 1, 5).

### 3.5 SUD-related suicide AAMR stratified by census region

**Figure 4:**
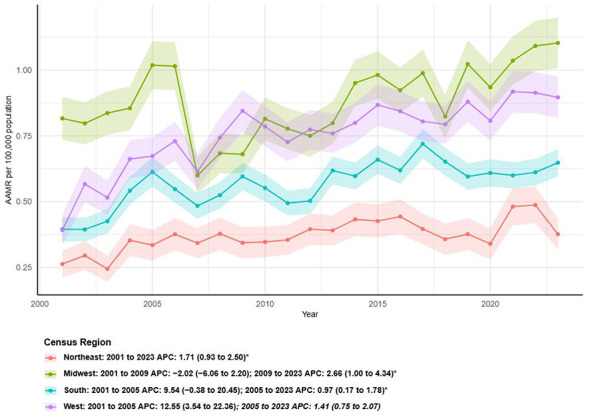
Census region-stratified SUD-related suicide AAMRs per 100,000 in the United States, 2001–2023. * Indicates that the annual percentage change (APC) is significantly different from zero at α = 0.05. AAMR, Age-adjusted mortality rates.

Across U.S. census regions, the Midwest had the highest AAMR (0.88), followed by the West (0.75), the South (0.56), and the Northeast with the lowest (0.37). In the West, AAMR increased steeply from 2001 to 2005 (APC: 12.55; 95% CI: 3.54–22.36), then stabilized with a modest but significant rise through 2023 (APC: 1.41; 95% CI: 0.75–2.07). The South followed a similar pattern, showing an early rise from 2001 to 2005 (APC: 9.54; 95% CI: −0.38 to 20.45), then continuing with a smaller but consistent increase (APC: 0.97; 95% CI: 0.17–1.78). In contrast, the Midwest exhibited a non-significant decline from 2001 to 2009 (APC: −2.02; 95% CI: −6.06 to 2.20), but subsequently shifted to a significant upward trend from 2009 to 2023 (APC: 2.66; 95% CI: 1.00–4.34), resulting in the highest recent rates of any region. The Northeast, while maintaining the lowest overall burden, showed a steady long-term increase (APC: 1.71; 95% CI: 0.93–2.50) without major fluctuations (Figure 4, Supplementary Tables 6, 7).

### 3.6 SUD-related suicide AAMR stratified by urbanization

**Figure 5:**
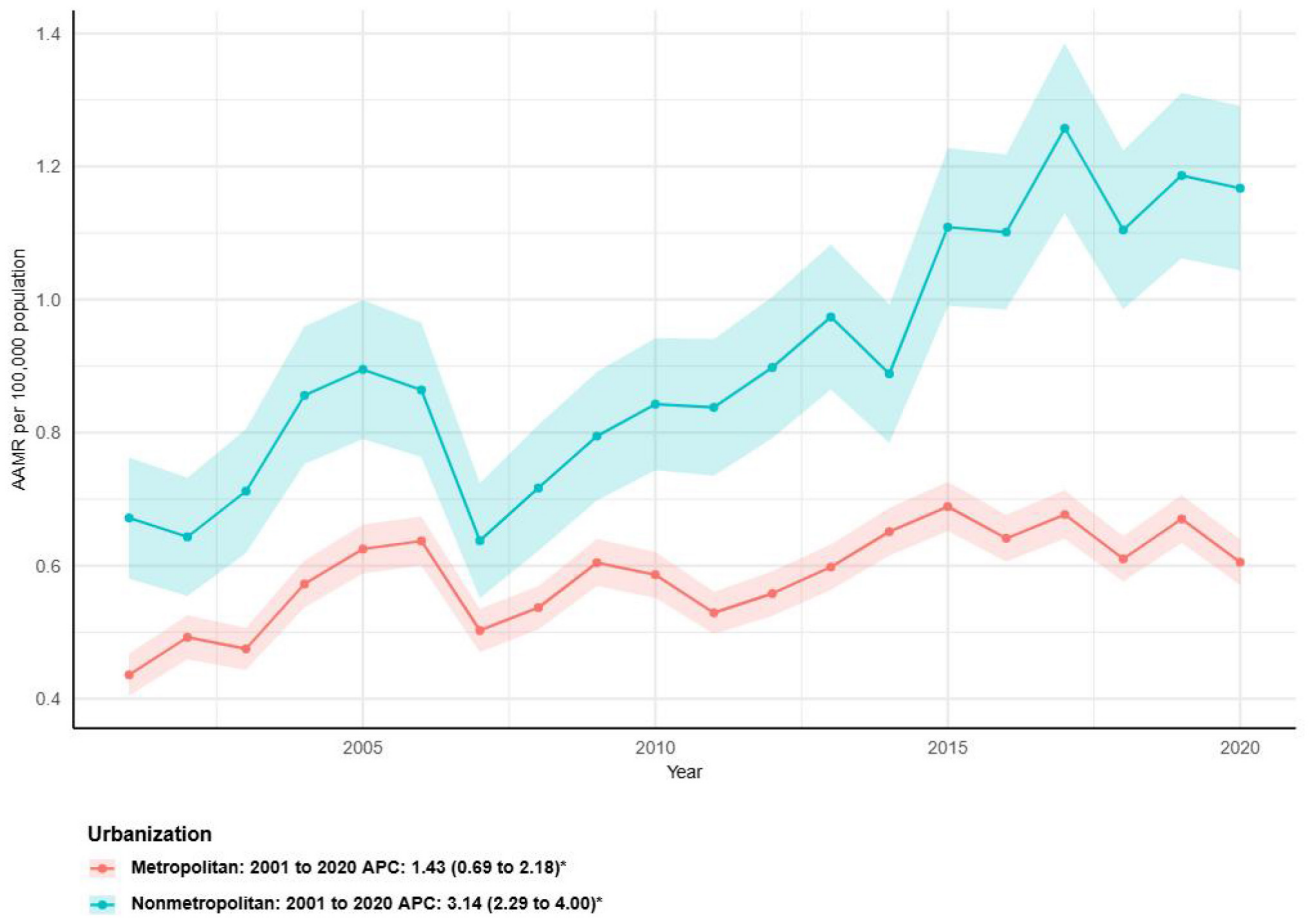
Urbanization-stratified SUD-related suicide AAMRs per 100,000 in the United States, 2001–2020. * Indicates that the annual percentage change (APC) is significantly different from zero at α = 0.05. AAMR, Age-adjusted mortality rates.

From 2001 to 2020, nonmetropolitan areas consistently had higher AAMRs than metropolitan areas (0.91 vs. 0.59). In nonmetropolitan areas, mortality rates increased modestly in the early 2000s, followed by a steeper and significant rise after 2010, reaching 1.17 in 2020 (APC: 3.14; 95% CI: 2.29–4.00). In metropolitan areas, the AAMR grew more gradually, rising from 0.46 in 2001 to 0.61 in 2020, with a slower but significant long-term increase (APC: 1.43; 95% CI: 0.69–2.18). These patterns widened the urban-rural gap across the available study period, with the disparity becoming most pronounced after 2011 (Figure 5, Supplementary Tables 6, 8).

### 3.7 Forecasting

Across the overall and sex-specific series, ADF testing supported nonstationarity (overall *p* = 0.09; female *p* = 0.99; male *p* = 0.19), and auto.arima() selected integrated models with one order of differencing. The overall series was best fit by ARIMA (0, 1, 0) (BIC −57.88), the female series by ARIMA (0, 1, 0) (BIC −71.80), and the male series by ARIMA (0, 1, 1) (BIC −43.12). In-sample performance was stable (RMSE 0.031–0.044; MAE 0.025–0.036; MAPE 3.73–5.06%). Residual diagnostics supported model adequacy, with low lag-1 autocorrelation (ACF1 −0.08 to −0.14) and nonsignificant Ljung-Box tests (overall *p* = 0.8406; female *p* = 0.6459; male *p* = 0.9320).

**Figure 6:**
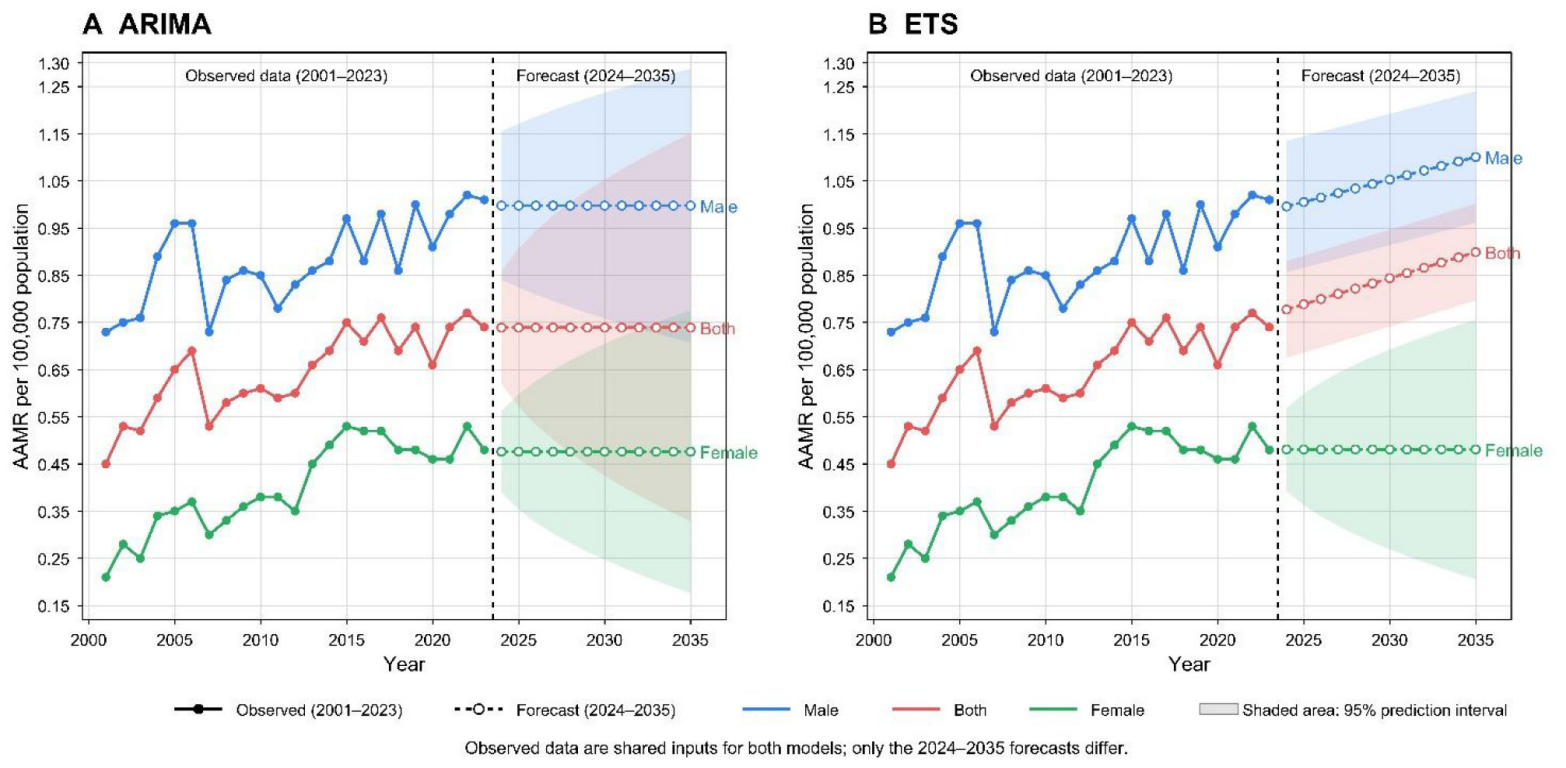
Forecasted age-adjusted mortality rates for substance use disorder–related suicide in the United States, overall and by sex, 2024–2035. Solid lines with filled circles represent the observed age-adjusted mortality rates from 2001 to 2023, which were used as common input data for both forecasting models. Dashed lines with open circles represent model-specific point forecasts from 2024 to 2035, and shaded areas indicate the corresponding 95% prediction intervals. **(A)** shows forecasts generated using the autoregressive integrated moving average model, and **(B)** shows forecasts generated using the error-trend-seasonality model. AAMR, age-adjusted mortality rate; ARIMA, autoregressive integrated moving average; ETS, error-trend-seasonality.

In the ARIMA framework, automated model selection favored integrated specifications without drift. Because such random-walk specifications produce multi-step point forecasts that converge to the last observed level, ARIMA point forecasts through 2035 were essentially flat, whereas prediction intervals widened progressively, indicating increasing uncertainty despite stable central projections. In the ETS sensitivity analysis, the overall and male series were best described by ETS (A, A, N), whereas the female series favored ETS (A, N, N). Model adequacy was supported by residual diagnostics (Ljung-Box: overall *p* = 0.1657; female *p* = 0.5033; male *p* = 0.7495). ETS projections suggested a modest upward trajectory for the overall series, with AAMR increasing to 0.90 in 2035, driven primarily by the male series, whereas the female series remained largely stable. Thus, the two approaches provided different forward-looking patterns and should be interpreted as model-specific projections rather than a single unified prediction of continued increase (Figure 6, Supplementary Table 9).

## 4 Discussion

This 23-year analysis of U.S. mortality data from the CDC WONDER database showed that SUD-related suicide mortality increased from 2001 to 2023 and varied substantially across demographic and geographic groups. Men had higher mortality than women, although the proportional increase among women was steeper. Non-Hispanic White individuals had the highest AAMR, whereas Non-Hispanic Black individuals had the lowest. Mortality also varied by age, with middle-aged adults experiencing the highest rates and the oldest age group examined, adults aged 65–74 years, showing notable increases. In addition, mortality was higher in the Midwest and in nonmetropolitan areas than in the Northeast and metropolitan areas. These observed patterns are consistent with, but do not prove, the potential influence of demographic, socioeconomic, behavioral, and structural factors on SUD-related suicide mortality. Forecasting analyses were model-dependent: ARIMA models projected stable central values with widening uncertainty, whereas ETS models suggested a modest upward trajectory through 2035.

Although SUD-related suicide mortality increased overall during the study period, a distinct sex disparity remained evident. From 2001 to 2023, males continuously showed higher AAMR than females. This disparity may reflect differences in substance use patterns, psychiatric comorbidity, help-seeking behavior, and the lethality of suicide methods, including firearms (22, 23), along with the established “gender paradox” in suicidal behavior (24). The reasons for these changing patterns cannot be determined from death-certificate data alone, but may include sex-specific changes in substance use behaviors and psychosocial stressors. For example, increases in alcohol misuse and prescription opioid consumption among middle-aged women may be associated with higher suicide risk in this group (25).

Variations in the age of initiation of substance use may also contribute to sex differences (26). Males often initiate alcohol or drug use at younger ages, which may function as a maladaptive coping mechanism for early-life trauma or stress and may be associated with earlier onset of severe substance use disorders (27, 28). Prior research suggests that early initiation of substance use is correlated with later SUDs and suicidal behaviors, particularly among males (29). These mechanisms are plausible but were not directly measured in the present study, and therefore should be interpreted as hypothesis-generating explanations rather than causal findings.

Our analysis also identified marked racial and ethnic differences in SUD-related suicide mortality. Non-Hispanic White individuals continuously showed the highest AAMR, whilst Non-Hispanic Black individuals exhibited the lowest rates, with Hispanic and other minority groups positioned in between. In 2023, the AAMR for Non-Hispanic White individuals was approximately fourfold higher than that of Non-Hispanic Black individuals, indicating a persistent descriptive disparity. These findings align with national data suggesting that SUDs are often reported at similar or lower prevalence among Black and Hispanic Americans compared with Non-Hispanic White populations (30). Recent analyses of trends in US life expectancy have predominantly concentrated on midlife fatalities resulting from suicide and substance-related poisonings (31), particularly among the White demographic (32). The widely recognized “deaths of despair” are thought to represent cohort-specific cumulative disadvantages associated with increasing misery, economic instability, and chronic pain within the White demographic (37). The opioid epidemic, fentanyl availability, and the economic effects of the Great Recession may have contributed to these patterns (33, 34), although these factors were not directly assessed in our analysis.

Distinct age-related disparities were also observed within the 15–74-year analytic population. In 2023, mortality rates were highest among middle-aged adults, particularly those aged 45–54 years, whereas younger adults had lower rates. At the same time, the 65–74-year group showed notable increases, with the AAPC reaching its highest value in this group. These findings suggest a dual pattern in which midlife remains the period of greatest absolute burden, while late adulthood before age 75 may represent an increasingly important high-risk period. Suicide deaths involving poisoning have previously peaked among individuals aged 45–54 years (35), corroborating the “deaths of despair” concept that associates increasing income disparity (36) and labor market deterioration (37) with midlife despair. The swift increase in mortality among adults aged 65–74 years may indicate the cumulative impact of prolonged opioid consumption, persistent pain, physiological susceptibility, and social isolation (38). Individuals aged 65–74 years may face heightened risks from opioids due to age-related alterations in drug metabolism and excretion, rendering them more vulnerable to side effects including drowsiness, cognitive impairment, falls, and injuries (39). Moreover, social isolation and loneliness are independently linked to deteriorating mental health, substance use, and increased mortality in later life (40).

From 2001 to 2020, rural areas of the United States consistently exhibited higher SUD-related suicide mortality than urban areas, and this rural–urban gap has widened over time. Numerous structural elements play a part in this discrepancy. Rural populations have less access to healthcare, and prompt treatment is hampered by a lack of medical and mental health professionals (41). These findings correspond with a previous study indicating elevated and swiftly increasing suicide rates in rural counties (42). Chronic rural poverty, limited access to education and employment, social isolation, and limited availability of addiction treatment may compound vulnerability to both SUD and suicide (43, 44). Evidence also suggests that rural patients may be less likely to receive office-based mental health care and may have different patterns of medication exposure, which could contribute to polysubstance involvement in some suicide deaths (45).

Geographic variation was also evident by census region. The Midwest continually demonstrated the greatest rates, succeeded by the West and South, while the Northeast retained the lowest rates. Contextual factors may contribute to these regional differences, such as restricted access to mental health and addiction services in rural regions (46), a high prevalence of methamphetamine and fentanyl usage in the Midwest and West (47), and persistent socioeconomic disadvantages in numerous Midwestern communities (48). Policy environments and firearm availability may also be relevant, because states with more stringent firearm regulations and stronger healthcare systems, particularly in the Northeast, have been reported to have lower firearm-related suicide mortality and better access to care (49). At the individual level, psychiatric illness, prior suicide attempts, stressful life events, and firearm availability are established risk factors for suicide (50). Macroeconomic factors, including poverty, unemployment, and educational disparities, are also associated with suicide risk (51).

This study has several limitations. First, reliance on CDC WONDER death-certificate data may lead to misclassification or underascertainment of SUD involvement or suicide as causes of death, which could affect mortality estimates. Second, the database lacks clinical details on SUD subtype, severity, psychiatric comorbidities, treatment history, method of suicide, toxicology findings, and social circumstances, limiting individual- level interpretation. Third, our case definition required suicide to be the underlying cause of death and SUD to be listed as a multiple/contributing cause; therefore, deaths in which SUD was the underlying cause and suicide appeared only as a contributing condition were not included. Fourth, as a retrospective ecological study using administrative mortality data, causal inference cannot be made. The possible mechanisms discussed above, including substance-related impulsivity, healthcare access, economic stressors, firearm access, and regional drug supply, were not directly measured and should be interpreted as hypothesis-generating. Fifth, forecasting results depend on model assumptions. ARIMA random-walk specifications produced flat point forecasts with widening prediction intervals, whereas ETS models suggested a modest increase; therefore, projections should be interpreted cautiously. Future research linking mortality data with individual-level clinical, behavioral, treatment, and socioeconomic data is needed to clarify drivers of SUD-related suicide mortality and guide targeted prevention initiatives.

## 5 Conclusion

This 23-year nationwide analysis showed that SUD-related suicide mortality in the United States increased from 2001 to 2023 and remained unevenly distributed by sex, race/ethnicity, age, region, and urbanization. Men, Non-Hispanic White individuals, middle-aged adults, nonmetropolitan residents, and the Midwest had the highest observed burden. Forecasts were model-dependent: ARIMA projected stable central values with widening uncertainty, whereas ETS suggested a modest increase through 2035, mainly among men. These findings support targeted public health strategies that improve access to mental health and addiction care and address high-burden populations and settings.

## Data Availability

The original contributions presented in the study are included in the article/Supplementary material, further inquiries can be directed to the corresponding author.
