# Supplementary material for: National trends, disparities and forecasts in substance use disorder–related suicide mortality in the United States: a CDC WONDER analysis
Source: Front Public Health. 2026 Jun 18;14:1830159. doi: 10.3389/fpubh.2026.1830159 (PMC13323130; doi:10.3389/fpubh.2026.1830159)
Supplement: Supplementary file 1 [file Data_Sheet_1.docx]

**Supplemental Table S1.** SUD and suicide-related deaths in the United States from 2001to 2023, stratified by gender and race.

| Year | Overall | Women | Men | NH Black | NH White | Hispanic | NH Other | Population |
| --- | --- | --- | --- | --- | --- | --- | --- | --- |
| 2001 | 961 | 223 | 738 | 43 | 858 | 37 | 21 | 207612586 |
| 2002 | 1058 | 287 | 771 | 58 | 930 | 45 | 22 | 209928491 |
| 2003 | 1085 | 264 | 821 | 38 | 970 | 37 | 37 | 212116669 |
| 2004 | 1290 | 362 | 928 | 52 | 1140 | 62 | 33 | 214617710 |
| 2005 | 1410 | 360 | 1050 | 48 | 1257 | 70 | 32 | 217229452 |
| 2006 | 1466 | 411 | 1055 | 48 | 1292 | 75 | 49 | 219902123 |
| 2007 | 1132 | 338 | 794 | 43 | 986 | 62 | 38 | 222422608 |
| 2008 | 1274 | 358 | 916 | 46 | 1131 | 64 | 31 | 224914640 |
| 2009 | 1418 | 451 | 967 | 51 | 1261 | 65 | 37 | 227293872 |
| 2010 | 1434 | 470 | 964 | 40 | 1271 | 63 | 54 | 228963770 |
| 2011 | 1370 | 466 | 904 | 42 | 1214 | 61 | 50 | 231478408 |
| 2012 | 1377 | 428 | 949 | 41 | 1217 | 63 | 49 | 233609978 |
| 2013 | 1543 | 541 | 1002 | 44 | 1365 | 69 | 56 | 235552408 |
| 2014 | 1634 | 591 | 1043 | 62 | 1423 | 82 | 51 | 237944180 |
| 2015 | 1764 | 629 | 1135 | 59 | 1548 | 100 | 49 | 240191698 |
| 2016 | 1730 | 667 | 1063 | 72 | 1478 | 107 | 67 | 241538579 |
| 2017 | 1793 | 627 | 1166 | 61 | 1557 | 106 | 65 | 243522393 |
| 2018 | 1650 | 609 | 1041 | 80 | 1411 | 105 | 47 | 244343113 |
| 2019 | 1784 | 580 | 1204 | 81 | 1507 | 114 | 78 | 245093847 |
| 2020 | 1635 | 558 | 1077 | 71 | 1344 | 145 | 74 | 246080730 |
| 2021 | 1820 | 591 | 1229 | 85 | 1497 | 134 | 75 | 249145244 |
| 2022 | 1868 | 636 | 1232 | 77 | 1514 | 166 | 73 | 249843757 |
| 2023 | 1859 | 612 | 1247 | 110 | 1517 | 141 | 66 | 250853337 |

**Supplemental Table S2.** SUD and suicide-related Age-Adjusted Mortality Rates per 100,000 in the United States, 2001-2023, overall and stratified by gender.

| Year | Overall | Female | Male |
| --- | --- | --- | --- |
| 2001 | 0.45 (0.42 to 0.47) | 0.21 (0.18 to 0.24) | 0.73 (0.68 to 0.79) |
| 2002 | 0.53 (0.50 to 0.56) | 0.28 (0.24 to 0.31) | 0.75 (0.69 to 0.80) |
| 2003 | 0.52 (0.49 to 0.55) | 0.25 (0.22 to 0.28) | 0.76 (0.71 to 0.81) |
| 2004 | 0.59 (0.56 to 0.63) | 0.34 (0.31 to 0.38) | 0.89 (0.83 to 0.94) |
| 2005 | 0.65 (0.61 to 0.68) | 0.35 (0.32 to 0.39) | 0.96 (0.90 to 1.01) |
| 2006 | 0.69 (0.65 to 0.72) | 0.37 (0.33 to 0.40) | 0.96 (0.90 to 1.02) |
| 2007 | 0.53 (0.49 to 0.56) | 0.30 (0.27 to 0.33) | 0.73 (0.68 to 0.78) |
| 2008 | 0.58 (0.55 to 0.61) | 0.33 (0.30 to 0.37) | 0.84 (0.78 to 0.89) |
| 2009 | 0.60 (0.57 to 0.64) | 0.36 (0.33 to 0.40) | 0.86 (0.80 to 0.91) |
| 2010 | 0.61 (0.58 to 0.65) | 0.38 (0.35 to 0.42) | 0.85 (0.79 to 0.90) |
| 2011 | 0.59 (0.56 to 0.62) | 0.38 (0.34 to 0.41) | 0.78 (0.73 to 0.83) |
| 2012 | 0.60 (0.56 to 0.63) | 0.35 (0.31 to 0.38) | 0.83 (0.77 to 0.88) |
| 2013 | 0.66 (0.62 to 0.69) | 0.45 (0.41 to 0.49) | 0.86 (0.80 to 0.91) |
| 2014 | 0.69 (0.65 to 0.72) | 0.49 (0.45 to 0.53) | 0.88 (0.83 to 0.94) |
| 2015 | 0.75 (0.71 to 0.78) | 0.53 (0.48 to 0.57) | 0.97 (0.91 to 1.03) |
| 2016 | 0.71 (0.68 to 0.74) | 0.52 (0.48 to 0.56) | 0.88 (0.83 to 0.94) |
| 2017 | 0.76 (0.72 to 0.79) | 0.52 (0.48 to 0.57) | 0.98 (0.93 to 1.04) |
| 2018 | 0.69 (0.65 to 0.72) | 0.48 (0.44 to 0.52) | 0.86 (0.81 to 0.91) |
| 2019 | 0.74 (0.70 to 0.77) | 0.48 (0.44 to 0.52) | 1.00 (0.94 to 1.06) |
| 2020 | 0.66 (0.63 to 0.70) | 0.46 (0.42 to 0.50) | 0.91 (0.85 to 0.96) |
| 2021 | 0.74 (0.70 to 0.77) | 0.46 (0.42 to 0.50) | 0.98 (0.93 to 1.04) |
| 2022 | 0.77 (0.74 to 0.81) | 0.53 (0.48 to 0.57) | 1.02 (0.96 to 1.07) |
| 2023 | 0.74 (0.70 to 0.77) | 0.48 (0.44 to 0.51) | 1.01 (0.96 to 1.07) |

**Supplemental Table S3.** Age-stratified SUD and suicide-related deaths in the United States from 2001to 2023.

| Year | 15-24 years | 25-34 years | 35-44 years | 45-54 years | 55-64 years | 65-74 years | Population |
| --- | --- | --- | --- | --- | --- | --- | --- |
| 2001 | 99 | 172 | 321 | 252 | 70 | 47 | 144833606 |
| 2002 | 113 | 183 | 336 | 290 | 100 | 36 | 146858382 |
| 2003 | 104 | 203 | 308 | 296 | 133 | 41 | 148846634 |
| 2004 | 128 | 253 | 333 | 380 | 150 | 46 | 150938828 |
| 2005 | 132 | 254 | 370 | 395 | 182 | 77 | 153292737 |
| 2006 | 154 | 242 | 371 | 416 | 216 | 67 | 155624180 |
| 2007 | 101 | 188 | 279 | 343 | 167 | 54 | 157690314 |
| 2008 | 97 | 183 | 326 | 411 | 192 | 65 | 159587617 |
| 2009 | 108 | 211 | 310 | 441 | 250 | 98 | 161383674 |
| 2010 | 111 | 216 | 296 | 458 | 273 | 80 | 162828035 |
| 2011 | 108 | 203 | 277 | 392 | 298 | 92 | 164802438 |
| 2012 | 97 | 211 | 290 | 440 | 252 | 87 | 166516716 |
| 2013 | 105 | 214 | 305 | 482 | 318 | 119 | 168240727 |
| 2014 | 116 | 262 | 330 | 426 | 371 | 129 | 170292776 |
| 2015 | 119 | 309 | 346 | 455 | 385 | 150 | 172416615 |
| 2016 | 128 | 273 | 326 | 415 | 424 | 164 | 173964174 |
| 2017 | 143 | 307 | 354 | 458 | 367 | 164 | 176104659 |
| 2018 | 110 | 306 | 320 | 375 | 392 | 147 | 177613416 |
| 2019 | 142 | 319 | 372 | 410 | 364 | 177 | 179040846 |
| 2020 | 129 | 339 | 335 | 349 | 313 | 170 | 180565367 |
| 2021 | 161 | 372 | 411 | 366 | 346 | 164 | 182743307 |
| 2022 | 141 | 346 | 430 | 391 | 383 | 177 | 184007299 |
| 2023 | 131 | 334 | 414 | 393 | 383 | 204 | 185987246 |

**Supplemental Table S4.** SUD and suicide-related Crude Mortality Rates per 100,000 in the United States, 2001-2023, stratified by age group.

| Year | 15-24 years | 25-34 years | 35-44 years | 45-54 years | 55-64 years | 65-74 years |
| --- | --- | --- | --- | --- | --- | --- |
| 2001 | 0.25 (0.20 to 0.30) | 0.44 (0.37 to 0.50) | 0.71 (0.63 to 0.79) | 0.64 (0.56 to 0.72) | 0.28 (0.22 to 0.35) | 0.26 (0.19 to 0.34) |
| 2002 | 0.28 (0.23 to 0.33) | 0.47 (0.40 to 0.53) | 0.75 (0.67 to 0.83) | 0.73 (0.64 to 0.81) | 0.37 (0.30 to 0.45) | 0.20 (0.14 to 0.27) |
| 2003 | 0.25 (0.20 to 0.30) | 0.52 (0.45 to 0.59) | 0.70 (0.62 to 0.78) | 0.73 (0.64 to 0.81) | 0.47 (0.39 to 0.56) | 0.22 (0.16 to 0.30) |
| 2004 | 0.31 (0.25 to 0.36) | 0.64 (0.56 to 0.72) | 0.76 (0.68 to 0.84) | 0.91 (0.82 to 1.00) | 0.51 (0.43 to 0.59) | 0.25 (0.18 to 0.33) |
| 2005 | 0.31 (0.26 to 0.36) | 0.65 (0.57 to 0.73) | 0.85 (0.76 to 0.94) | 0.93 (0.84 to 1.02) | 0.59 (0.51 to 0.68) | 0.41 (0.32 to 0.51) |
| 2006 | 0.36 (0.30 to 0.42) | 0.61 (0.54 to 0.69) | 0.86 (0.77 to 0.95) | 0.96 (0.87 to 1.05) | 0.68 (0.59 to 0.77) | 0.35 (0.27 to 0.44) |
| 2007 | 0.23 (0.19 to 0.28) | 0.47 (0.41 to 0.54) | 0.65 (0.58 to 0.73) | 0.78 (0.70 to 0.86) | 0.50 (0.43 to 0.58) | 0.27 (0.21 to 0.36) |
| 2008 | 0.22 (0.18 to 0.27) | 0.46 (0.39 to 0.52) | 0.77 (0.69 to 0.86) | 0.92 (0.84 to 1.01) | 0.56 (0.48 to 0.64) | 0.32 (0.24 to 0.40) |
| 2009 | 0.25 (0.20 to 0.29) | 0.52 (0.45 to 0.59) | 0.75 (0.66 to 0.83) | 0.98 (0.89 to 1.07) | 0.71 (0.62 to 0.79) | 0.46 (0.37 to 0.56) |
| 2010 | 0.25 (0.21 to 0.30) | 0.53 (0.46 to 0.60) | 0.72 (0.64 to 0.80) | 1.02 (0.92 to 1.11) | 0.75 (0.66 to 0.84) | 0.37 (0.29 to 0.46) |
| 2011 | 0.25 (0.20 to 0.29) | 0.49 (0.42 to 0.55) | 0.68 (0.60 to 0.76) | 0.88 (0.79 to 0.96) | 0.78 (0.69 to 0.87) | 0.41 (0.33 to 0.50) |
| 2012 | 0.22 (0.18 to 0.27) | 0.50 (0.43 to 0.57) | 0.72 (0.63 to 0.80) | 0.99 (0.90 to 1.09) | 0.65 (0.57 to 0.73) | 0.36 (0.29 to 0.45) |
| 2013 | 0.24 (0.19 to 0.28) | 0.50 (0.43 to 0.57) | 0.75 (0.67 to 0.84) | 1.10 (1.00 to 1.20) | 0.81 (0.72 to 0.90) | 0.47 (0.39 to 0.56) |
| 2014 | 0.26 (0.22 to 0.31) | 0.60 (0.53 to 0.67) | 0.81 (0.73 to 0.90) | 0.98 (0.89 to 1.07) | 0.93 (0.83 to 1.02) | 0.49 (0.40 to 0.57) |
| 2015 | 0.27 (0.22 to 0.32) | 0.70 (0.62 to 0.78) | 0.85 (0.76 to 0.94) | 1.05 (0.96 to 1.15) | 0.94 (0.85 to 1.04) | 0.54 (0.46 to 0.63) |
| 2016 | 0.29 (0.24 to 0.35) | 0.61 (0.54 to 0.68) | 0.81 (0.72 to 0.89) | 0.97 (0.88 to 1.06) | 1.02 (0.93 to 1.12) | 0.57 (0.49 to 0.66) |
| 2017 | 0.33 (0.28 to 0.38) | 0.68 (0.60 to 0.75) | 0.87 (0.78 to 0.96) | 1.08 (0.98 to 1.18) | 0.87 (0.78 to 0.96) | 0.55 (0.47 to 0.64) |
| 2018 | 0.26 (0.21 to 0.30) | 0.67 (0.59 to 0.74) | 0.78 (0.69 to 0.86) | 0.90 (0.81 to 0.99) | 0.93 (0.84 to 1.02) | 0.48 (0.40 to 0.56) |
| 2019 | 0.33 (0.28 to 0.39) | 0.69 (0.62 to 0.77) | 0.89 (0.80 to 0.98) | 1.00 (0.91 to 1.10) | 0.86 (0.77 to 0.95) | 0.56 (0.48 to 0.65) |
| 2020 | 0.30 (0.25 to 0.36) | 0.74 (0.66 to 0.81) | 0.80 (0.71 to 0.88) | 0.86 (0.77 to 0.96) | 0.74 (0.66 to 0.82) | 0.52 (0.44 to 0.60) |
| 2021 | 0.37 (0.32 to 0.43) | 0.82 (0.73 to 0.90) | 0.95 (0.86 to 1.04) | 0.90 (0.81 to 0.99) | 0.81 (0.72 to 0.89) | 0.49 (0.41 to 0.56) |
| 2022 | 0.32 (0.27 to 0.37) | 0.76 (0.68 to 0.84) | 0.98 (0.89 to 1.08) | 0.97 (0.87 to 1.06) | 0.91 (0.82 to 1.00) | 0.52 (0.45 to 0.60) |
| 2023 | 0.30 (0.25 to 0.35) | 0.73 (0.65 to 0.81) | 0.93 (0.84 to 1.02) | 0.97 (0.87 to 1.07) | 0.92 (0.82 to 1.01) | 0.59 (0.51 to 0.67) |

**Supplemental Table S5.** SUD and suicide-related Age-Adjusted Mortality Rates per 100,000 in the United States, 2001-2023, stratified by race/ethnicity.

| Year | Hispanic | NH Black | NH Other | NH White |
| --- | --- | --- | --- | --- |
| 2001 | 0.13 (0.09 to 0.19) | 0.18 (0.13 to 0.24) | 0.19 (0.12 to 0.30) | 0.58 (0.54 to 0.62) |
| 2002 | 0.18 (0.12 to 0.24) | 0.23 (0.17 to 0.29) | 0.16 (0.10 to 0.24) | 0.66 (0.62 to 0.70) |
| 2003 | 0.13 (0.09 to 0.18) | 0.14 (0.10 to 0.20) | 0.30 (0.21 to 0.41) | 0.65 (0.61 to 0.69) |
| 2004 | 0.19 (0.14 to 0.24) | 0.21 (0.16 to 0.28) | 0.30 (0.21 to 0.42) | 0.77 (0.73 to 0.82) |
| 2005 | 0.25 (0.19 to 0.32) | 0.18 (0.13 to 0.24) | 0.26 (0.17 to 0.37) | 0.84 (0.80 to 0.89) |
| 2006 | 0.25 (0.19 to 0.32) | 0.19 (0.14 to 0.26) | 0.38 (0.28 to 0.51) | 0.87 (0.82 to 0.91) |
| 2007 | 0.19 (0.14 to 0.24) | 0.17 (0.12 to 0.23) | 0.28 (0.20 to 0.39) | 0.68 (0.63 to 0.72) |
| 2008 | 0.20 (0.15 to 0.26) | 0.19 (0.13 to 0.25) | 0.22 (0.15 to 0.31) | 0.76 (0.71 to 0.81) |
| 2009 | 0.19 (0.15 to 0.24) | 0.17 (0.12 to 0.22) | 0.26 (0.18 to 0.36) | 0.84 (0.79 to 0.89) |
| 2010 | 0.19 (0.14 to 0.25) | 0.15 (0.11 to 0.21) | 0.37 (0.28 to 0.48) | 0.85 (0.80 to 0.89) |
| 2011 | 0.18 (0.14 to 0.24) | 0.13 (0.10 to 0.18) | 0.35 (0.26 to 0.47) | 0.78 (0.73 to 0.82) |
| 2012 | 0.19 (0.14 to 0.24) | 0.13 (0.09 to 0.18) | 0.30 (0.22 to 0.40) | 0.79 (0.74 to 0.83) |
| 2013 | 0.20 (0.15 to 0.26) | 0.13 (0.10 to 0.18) | 0.35 (0.26 to 0.45) | 0.89 (0.84 to 0.94) |
| 2014 | 0.22 (0.18 to 0.28) | 0.21 (0.16 to 0.27) | 0.29 (0.22 to 0.39) | 0.94 (0.89 to 0.99) |
| 2015 | 0.24 (0.20 to 0.29) | 0.20 (0.15 to 0.26) | 0.26 (0.19 to 0.34) | 1.00 (0.95 to 1.06) |
| 2016 | 0.25 (0.21 to 0.30) | 0.23 (0.18 to 0.30) | 0.39 (0.30 to 0.49) | 0.93 (0.88 to 0.98) |
| 2017 | 0.25 (0.20 to 0.30) | 0.20 (0.15 to 0.26) | 0.38 (0.30 to 0.49) | 1.03 (0.97 to 1.08) |
| 2018 | 0.25 (0.20 to 0.30) | 0.26 (0.20 to 0.32) | 0.28 (0.20 to 0.37) | 0.91 (0.86 to 0.96) |
| 2019 | 0.27 (0.22 to 0.32) | 0.28 (0.22 to 0.35) | 0.41 (0.33 to 0.52) | 1.03 (0.98 to 1.09) |
| 2020 | 0.32 (0.27 to 0.37) | 0.24 (0.19 to 0.31) | 0.42 (0.33 to 0.52) | 0.91 (0.86 to 0.96) |
| 2021 | 0.30 (0.25 to 0.35) | 0.30 (0.23 to 0.37) | 0.44 (0.34 to 0.55) | 1.04 (0.99 to 1.10) |
| 2022 | 0.32 (0.27 to 0.37) | 0.25 (0.20 to 0.31) | 0.42 (0.33 to 0.53) | 1.03 (0.97 to 1.08) |
| 2023 | 0.31 (0.26 to 0.36) | 0.37 (0.30 to 0.44) | 0.33 (0.25 to 0.42) | 1.02 (0.96 to 1.07) |

**Supplemental Table S6.** SUD and suicide-related deaths in the United States from 2001to 2023, stratified by region.

| Year | Northeast | Midwest | South | West | Metropolitan | Nonmetropolitan |
| --- | --- | --- | --- | --- | --- | --- |
| 2001 | 104 | 382 | 287 | 188 | 749 | 212 |
| 2002 | 113 | 384 | 307 | 254 | 853 | 205 |
| 2003 | 100 | 397 | 333 | 255 | 858 | 227 |
| 2004 | 131 | 414 | 410 | 335 | 1021 | 269 |
| 2005 | 131 | 474 | 464 | 341 | 1121 | 289 |
| 2006 | 152 | 490 | 446 | 378 | 1174 | 292 |
| 2007 | 140 | 288 | 383 | 321 | 915 | 217 |
| 2008 | 150 | 316 | 431 | 377 | 1044 | 230 |
| 2009 | 136 | 343 | 500 | 439 | 1147 | 271 |
| 2010 | 146 | 401 | 464 | 423 | 1141 | 293 |
| 2011 | 158 | 396 | 434 | 382 | 1093 | 277 |
| 2012 | 177 | 364 | 422 | 414 | 1087 | 290 |
| 2013 | 186 | 400 | 545 | 412 | 1215 | 328 |
| 2014 | 181 | 469 | 539 | 445 | 1327 | 307 |
| 2015 | 190 | 486 | 607 | 481 | 1402 | 362 |
| 2016 | 191 | 473 | 585 | 481 | 1355 | 375 |
| 2017 | 183 | 497 | 649 | 464 | 1393 | 400 |
| 2018 | 152 | 418 | 610 | 470 | 1290 | 360 |
| 2019 | 162 | 513 | 581 | 528 | 1406 | 378 |
| 2020 | 139 | 453 | 563 | 480 | 1266 | 369 |
| 2021 | 188 | 514 | 584 | 534 |  |  |
| 2022 | 205 | 529 | 588 | 546 |  |  |
| 2023 | 173 | 543 | 624 | 519 |  |  |

**Supplemental Table S7.** SUD and suicide-related Age-Adjusted Mortality Rates per 100,000 in the United States, 2001-2023, stratified by region.

| Year | Midwest | Northeast | South | West |
| --- | --- | --- | --- | --- |
| 2001 | 0.82 (0.73 to 0.90) | 0.26 (0.21 to 0.31) | 0.39 (0.35 to 0.44) | 0.39 (0.34 to 0.45) |
| 2002 | 0.80 (0.72 to 0.88) | 0.30 (0.24 to 0.35) | 0.39 (0.35 to 0.44) | 0.57 (0.50 to 0.64) |
| 2003 | 0.84 (0.75 to 0.92) | 0.24 (0.20 to 0.29) | 0.43 (0.38 to 0.47) | 0.52 (0.45 to 0.58) |
| 2004 | 0.85 (0.77 to 0.94) | 0.35 (0.29 to 0.41) | 0.54 (0.49 to 0.59) | 0.66 (0.59 to 0.73) |
| 2005 | 1.02 (0.93 to 1.11) | 0.33 (0.28 to 0.39) | 0.61 (0.56 to 0.67) | 0.67 (0.60 to 0.74) |
| 2006 | 1.01 (0.92 to 1.10) | 0.38 (0.32 to 0.44) | 0.55 (0.50 to 0.60) | 0.73 (0.66 to 0.80) |
| 2007 | 0.60 (0.53 to 0.67) | 0.34 (0.29 to 0.40) | 0.48 (0.44 to 0.53) | 0.61 (0.55 to 0.68) |
| 2008 | 0.68 (0.61 to 0.76) | 0.38 (0.32 to 0.44) | 0.52 (0.47 to 0.57) | 0.74 (0.67 to 0.82) |
| 2009 | 0.68 (0.61 to 0.75) | 0.34 (0.28 to 0.40) | 0.60 (0.54 to 0.65) | 0.84 (0.77 to 0.92) |
| 2010 | 0.81 (0.73 to 0.90) | 0.35 (0.29 to 0.40) | 0.55 (0.50 to 0.60) | 0.79 (0.71 to 0.86) |
| 2011 | 0.78 (0.70 to 0.85) | 0.35 (0.30 to 0.41) | 0.49 (0.45 to 0.54) | 0.73 (0.65 to 0.80) |
| 2012 | 0.75 (0.67 to 0.83) | 0.40 (0.34 to 0.46) | 0.50 (0.45 to 0.55) | 0.77 (0.70 to 0.85) |
| 2013 | 0.80 (0.72 to 0.88) | 0.39 (0.33 to 0.45) | 0.62 (0.56 to 0.67) | 0.76 (0.69 to 0.83) |
| 2014 | 0.95 (0.86 to 1.04) | 0.43 (0.37 to 0.50) | 0.60 (0.55 to 0.65) | 0.80 (0.72 to 0.87) |
| 2015 | 0.98 (0.89 to 1.07) | 0.43 (0.36 to 0.49) | 0.66 (0.61 to 0.71) | 0.87 (0.79 to 0.95) |
| 2016 | 0.92 (0.84 to 1.01) | 0.44 (0.38 to 0.51) | 0.62 (0.57 to 0.67) | 0.84 (0.77 to 0.92) |
| 2017 | 0.99 (0.90 to 1.08) | 0.40 (0.34 to 0.46) | 0.72 (0.66 to 0.78) | 0.80 (0.73 to 0.88) |
| 2018 | 0.82 (0.74 to 0.90) | 0.36 (0.30 to 0.42) | 0.65 (0.60 to 0.70) | 0.79 (0.72 to 0.87) |
| 2019 | 1.02 (0.93 to 1.11) | 0.38 (0.32 to 0.44) | 0.60 (0.55 to 0.65) | 0.88 (0.80 to 0.96) |
| 2020 | 0.93 (0.85 to 1.02) | 0.34 (0.28 to 0.40) | 0.61 (0.56 to 0.66) | 0.81 (0.73 to 0.88) |
| 2021 | 1.04 (0.94 to 1.13) | 0.48 (0.41 to 0.55) | 0.60 (0.55 to 0.65) | 0.92 (0.84 to 1.00) |
| 2022 | 1.09 (1.00 to 1.19) | 0.49 (0.42 to 0.56) | 0.61 (0.56 to 0.66) | 0.91 (0.83 to 0.99) |
| 2023 | 1.10 (1.01 to 1.20) | 0.38 (0.32 to 0.43) | 0.65 (0.60 to 0.70) | 0.90 (0.82 to 0.97) |

**Supplemental Table S8.** SUD and suicide-related Age-Adjusted Mortality Rates per 100,000 in the United States, 2001-2020, stratified by metropolitan/nonmetropolitan.

| Year | Metropolitan | Nonmetropolitan |
| --- | --- | --- |
| 2001 | 0.44 (0.40 to 0.47) | 0.67 (0.58 to 0.76) |
| 2002 | 0.49 (0.46 to 0.53) | 0.64 (0.55 to 0.73) |
| 2003 | 0.47 (0.44 to 0.51) | 0.71 (0.62 to 0.81) |
| 2004 | 0.57 (0.54 to 0.61) | 0.86 (0.75 to 0.96) |
| 2005 | 0.63 (0.59 to 0.66) | 0.89 (0.79 to 1.00) |
| 2006 | 0.64 (0.60 to 0.67) | 0.86 (0.76 to 0.97) |
| 2007 | 0.50 (0.47 to 0.54) | 0.64 (0.55 to 0.72) |
| 2008 | 0.54 (0.50 to 0.57) | 0.72 (0.62 to 0.81) |
| 2009 | 0.60 (0.57 to 0.64) | 0.79 (0.70 to 0.89) |
| 2010 | 0.59 (0.55 to 0.62) | 0.84 (0.74 to 0.94) |
| 2011 | 0.53 (0.50 to 0.56) | 0.84 (0.74 to 0.94) |
| 2012 | 0.56 (0.52 to 0.59) | 0.90 (0.79 to 1.00) |
| 2013 | 0.60 (0.56 to 0.63) | 0.97 (0.86 to 1.08) |
| 2014 | 0.65 (0.62 to 0.69) | 0.89 (0.78 to 0.99) |
| 2015 | 0.69 (0.65 to 0.73) | 1.11 (0.99 to 1.23) |
| 2016 | 0.64 (0.61 to 0.68) | 1.10 (0.98 to 1.22) |
| 2017 | 0.68 (0.64 to 0.71) | 1.26 (1.13 to 1.39) |
| 2018 | 0.61 (0.58 to 0.64) | 1.10 (0.99 to 1.22) |
| 2019 | 0.67 (0.63 to 0.71) | 1.19 (1.06 to 1.31) |
| 2020 | 0.61 (0.57 to 0.64) | 1.17 (1.04 to 1.29) |

**Supplemental Table S9.** Using ARIMA and ETS models to forecast the age-adjusted mortality rates for SUD-related suicide from 2024 to 2035, stratified by total and sex group stratified.

| Measure | ADF_p | Model | AIC | AICc | BIC | RMSE | MAE | MAPE | ACF1 | LjungBox_Q | LjungBox_p |
| --- | --- | --- | --- | --- | --- | --- | --- | --- | --- | --- | --- |
| ARIMA | | | | | | | | | | | |
| Overall | 0.0908 | (0,1,0) | -58.97 | -58.77 | -57.88 | 0.0592 | 0.0487 | 7.68 | -0.3659 | 12.4002 | 0.2592 |
| Female | 0.4207 | (0,1,0) | -72.9 | -72.7 | -71.8 | 0.0431 | 0.0328 | 8.55 | -0.2185 | 12.2223 | 0.2705 |
| Male | 0.0818 | (0,1,1) | -45.3 | -44.67 | -43.12 | 0.0766 | 0.0589 | 6.64 | -0.0943 | 7.597 | 0.5752 |
| ETS | | | | | | | | | | | |
| Overall | 0.090809 | (A,A,N) | / | -54.4851 | -52.337041 | 0.047533 | 0.03786 | 6.088504 | 0.194424 | 14.162886 | 0.165691 |
| Female | 0.420718 | (A,N,N) | / | -65.499293 | -63.355968 | 0.042872 | 0.033279 | 8.669851 | -0.130726 | 9.306201 | 0.503311 |
| Male | 0.081847 | (A,A,N) | / | -40.424184 | -38.276124 | 0.064528 | 0.053204 | 6.09338 | 0.107687 | 6.742732 | 0.749489 |
