# Supplementary material for: National trends, disparities and forecasts in substance use disorder–related suicide mortality in the United States: a CDC WONDER analysis
Source: Front Public Health. 2026 Jun 18;14:1830159. doi: 10.3389/fpubh.2026.1830159 (PMC13323130; doi:10.3389/fpubh.2026.1830159)
Supplement: Supplementary file 2 [file Table_1.docx]

STROBE Statement—checklist of items that should be included in reports of observational studies

|  | Item No. | Recommendation | Reported in section | Relevant text from manuscript |
| --- | --- | --- | --- | --- |
| **Title and abstract** | 1 | (*a*) Indicate the study’s design with a commonly used term in the title or the abstract | Title and abstract | National Trends, Disparities and Forecasts in Substance Use Disorder–Related Suicide Mortality in the United States: A CDC WONDER Analysis: A Comprehensive Analysis of Demographic and Geographic Variations. The abstract specifies it is a national trend analysis using CDC WONDER data. |
|  |  | (*b*) Provide in the abstract an informative and balanced summary of what was done and what was found | Abstract | Abstract provides objectives, methods (death certificate data, Joinpoint regression), results and conclusions. |
| Introduction | | | |  |
| Background/rationale | 2 | Explain the scientific background and rationale for the investigation being reported | Introduction | Introduction explains that suicide is a leading cause of death and that substance use disorders (SUDs) are major risk factors for suicide. For example, it notes that globally suicide rates have declined but US rates rose by 33% from 1999 to 2017 (14 to 14 per 100k). It cites evidence that roughly 25–50% of suicides involve SUDs. It emphasizes that national trends in SUD-related suicide mortality and disparities have not been well studied. This motivates a comprehensive investigation using nationwide data. |
| Objectives | 3 | State specific objectives, including any prespecified hypotheses | Introduction | To examine national trends in SUD-related suicide mortality from 2001–2023 and identify demographic and geographic disparities. Examine mortality trends…from 2001 to 2023, categorized by age, sex, race, and geographic region, to pinpoint high-risk populations. |
| Methods | | | |  |
| Study design | 4 | Present key elements of study design early in the paper | Methods | Methods: Nationwide, population-based observational study using CDC WONDER death certificate data (2001–2023). |
| Setting | 5 | Describe the setting, locations, and relevant dates, including periods of recruitment, exposure, follow-up, and data collection | Methods | The setting is the United States over the years 2001–2023. All analyses were based on U.S. death certificate data obtained from the CDC WONDER database, which includes underlying cause of death and demographics for US residents. |
| Participants | 6 | (*a*) *Cohort study*—Give the eligibility criteria, and the sources and methods of selection of participants. Describe methods of follow-up  *Case-control study*—Give the eligibility criteria, and the sources and methods of case ascertainment and control selection. Give the rationale for the choice of cases and controls  *Cross-sectional study*—Give the eligibility criteria, and the sources and methods of selection of participants | Methods | All death records in CDC WONDER (2001–2023) with ICD-10 codes for SUD (F10–F19) and suicide (U03, X60–X84, Y87.0). |
|  |  | (*b*) *Cohort study*—For matched studies, give matching criteria and number of exposed and unexposed  *Case-control study*—For matched studies, give matching criteria and the number of controls per case |  | Not applicable |
| Variables | 7 | Clearly define all outcomes, exposures, predictors, potential confounders, and effect modifiers. Give diagnostic criteria, if applicable | Methods | Death from suicide in individuals with SUD (defined by ICD-10 codes as above). Predictor (exposure) variables include demographic and geographic factors: sex, age group, race/ethnicity, Census region, and urban-rural status. Age was categorized into groups (15–24, 25–34, 35–44, 45–54, 55–64, 65–74). Race/ethnicity categories are Hispanic, Non-Hispanic (NH) Black, NH White, and NH Other (including NH Asian/Pacific Islander, etc). Census regions are Northeast, Midwest, South, West. Urban-rural classification followed the NCHS scheme (large/medium/small metro vs rural). No other exposures, confounders, or effect modifiers were defined beyond these standard demographic covariates. |
| Data sources/ measurement | 8* | For each variable of interest, give sources of data and details of methods of assessment (measurement). Describe comparability of assessment methods if there is more than one group | Methods | Source: CDC WONDER death certificate data. ICD-10 coding used to define outcomes. Demographics from certificates. Standardized mortality rates. |
| Bias | 9 | Describe any efforts to address potential sources of bias | Methods | Public death certificate data; standardized definitions; age-adjusted rates. Limitation acknowledged: possible misclassification of cause of death. |
| Study size | 10 | Explain how the study size was arrived at | Methods | The study included all identified SUD-related suicide deaths in the U.S. from 2001 to 2023. The authors did not sample or calculate a target sample size; rather the size is determined by the census of death records meeting inclusion criteria. |

Continued on next page

| Quantitative variables | 11 | Explain how quantitative variables were handled in the analyses. If applicable, describe which groupings were chosen and why | Methods | Quantitative variables were handled as follows: Age was analyzed in predefined categorical groups (15–24, 25–34, …, 65–74). Mortality rates were expressed as age-adjusted rates per 100,000 using the 2000 standard population. Trends over time were assessed with Joinpoint regression. |
| --- | --- | --- | --- | --- |
| Statistical methods | 12 | (*a*) Describe all statistical methods, including those used to control for confounding | Methods | Joinpoint regression to estimate annual percent change (APC) and average annual percent change (AAPC); log-linear models; p<0.05 significance. |
|  |  | (*b*) Describe any methods used to examine subgroups and interactions | Methods | Perform subgroup analyses by year, gender, race, region, age group, etc. |
|  |  | (*c*) Explain how missing data were addressed |  |  |
|  |  | (*d*) *Cohort study*—If applicable, explain how loss to follow-up was addressed  *Case-control study*—If applicable, explain how matching of cases and controls was addressed  *Cross-sectional study*—If applicable, describe analytical methods taking account of sampling strategy |  | Not applicable |
|  |  | (*e*) Describe any sensitivity analyses | Methods | Subgroup analysis |
| Results | | | | |
| Participants | 13* | (a) Report numbers of individuals at each stage of study—eg numbers potentially eligible, examined for eligibility, confirmed eligible, included in the study, completing follow-up, and analysed | Results | Total deaths: 34,355. Table 1 shows counts by year, sex, race, region, age groups. The supplementary materials contain all data. |
|  |  | (b) Give reasons for non-participation at each stage |  |  |
|  |  | (c) Consider use of a flow diagram |  | Please refer to the graphic summary. |
| Descriptive data | 14* | (a) Give characteristics of study participants (eg demographic, clinical, social) and information on exposures and potential confounders | Results | Descriptive data provided in Table 1 and supplementary materials: deaths by sex, race/ethnicity, age group, region, urbanization. |
|  |  | (b) Indicate number of participants with missing data for each variable of interest |  | Not applicable |
|  |  | (c) *Cohort study*—Summarise follow-up time (eg, average and total amount) |  | Not applicable |
| Outcome data | 15* | *Cohort study*—Report numbers of outcome events or summary measures over time | Results | Between 2001 and 2023, the overall AAMR for SUD-related suicides increased from 0.45 to 0.74, representing a significant long-term rise (AAPC: 1.70; 95% CI: 1.16–2.24). The trend was characterized by a sharp increase in the early 2000s (APC: 6.12; 95% CI: 2.84–9.52 for 2001–2005), followed by a period of relative stability through the late 2000s and mid-2010s (APC: 0.47; 95% CI: –0.65 to 1.59 for 2005–2015), and a renewed significant incline thereafter (APC: 2.29; 95% CI: 1.05–3.55 for 2015–2023), culminating in nearly double the annual deaths by the end of the study period |
|  |  | *Case-control study—*Report numbers in each exposure category, or summary measures of exposure |  | Not applicable |
|  |  | *Cross-sectional study—*Report numbers of outcome events or summary measures |  | Not applicable |
| Main results | 16 | (*a*) Give unadjusted estimates and, if applicable, confounder-adjusted estimates and their precision (eg, 95% confidence interval). Make clear which confounders were adjusted for and why they were included | Results | All results reported as age-adjusted mortality rates per 100,000 with 95% CI; stratified by demographic categories. All data are available in the results and supplementary materials. |
|  |  | (*b*) Report category boundaries when continuous variables were categorized |  | Not applicable |
|  |  | (*c*) If relevant, consider translating estimates of relative risk into absolute risk for a meaningful time period |  | Not applicable |

Continued on next page

| Other analyses | 17 | Report other analyses done—eg analyses of subgroups and interactions, and sensitivity analyses | Results | Subgroup analyses by sex, age, race/ethnicity, region, urban/rural; joinpoint identified trend shifts. |
| --- | --- | --- | --- | --- |
| Discussion | | | | |
| Key results | 18 | Summarise key results with reference to study objectives | Discussion | The increasing public health burden of SUD was highlighted by the consistent increase in suicide mortality linked to it from 2001 to 2023 in this 23-year analysis of U.S. mortality data from the CDC WONDER database. Patterns of mortality varied among populations. Rates were generally higher for men than for women; however, the gender difference decreased as female mortality increased more rapidly. Non-Hispanic White people were the most burdened by race and ethnicity, while non-Hispanic Black people had the lowest rates. Mortality rates also varied by age, with middle-aged individuals suffering the highest rates and older groups showing the steepest increases. Furthermore, mortality was consistently greater among Midwest and rural individuals than among urban and Northeast residents. |
| Limitations | 19 | Discuss limitations of the study, taking into account sources of potential bias or imprecision. Discuss both direction and magnitude of any potential bias | Discussion | The last paragraph of the discussion section. |
| Interpretation | 20 | Give a cautious overall interpretation of results considering objectives, limitations, multiplicity of analyses, results from similar studies, and other relevant evidence | Discussion | These results highlight the interplay of demographic, socioeconomic, and structural determinants in SUD-related suicide mortality, underscoring the necessity of focused preventative and policy measures. |
| Generalisability | 21 | Discuss the generalisability (external validity) of the study results | Discussion | The last paragraph of the discussion section. |
| Other information | |  | | |
| Funding | 22 | Give the source of funding and the role of the funders for the present study and, if applicable, for the original study on which the present article is based | Funding | Detailed information can be found in the funding section. |

*Give information separately for cases and controls in case-control studies and, if applicable, for exposed and unexposed groups in cohort and cross-sectional studies.

**Note:** An Explanation and Elaboration article discusses each checklist item and gives methodological background and published examples of transparent reporting. The STROBE checklist is best used in conjunction with this article (freely available on the Web sites of PLoS Medicine at http://www.plosmedicine.org/, Annals of Internal Medicine at http://www.annals.org/, and Epidemiology at http://www.epidem.com/). Information on the STROBE Initiative is available at www.strobe-statement.org.
